# Associations of red and processed meat intake with screen-detected colorectal lesions

**DOI:** 10.1017/S0007114522002860

**Published:** 2023-06-28

**Authors:** Ane Sørlie Kværner, Einar Birkeland, Elina Vinberg, Geir Hoff, Anette Hjartåker, Trine B. Rounge, Paula Berstad

**Affiliations:** 1 Section for Colorectal Cancer Screening, Cancer Registry of Norway, Oslo, Norway; 2 Department of Research, Cancer Registry of Norway, Oslo, Norway; 3 Department of Informatics, University of Oslo, Oslo, Norway; 4 Department of Research, Telemark Hospital, Skien, Norway; 5 Department of Nutrition, University of Oslo, Oslo, Norway; 6 Department of Pharmacy, University of Oslo, Oslo, Norway

**Keywords:** Colorectal cancer, Advanced colorectal lesions, Screening, Fecal immunochemical test, Meat, Red meat, Processed meat

## Abstract

Limited data exist regarding the role of meat consumption in early-stage colorectal carcinogenesis. We examined associations of red and processed meat intake with screen-detected colorectal lesions in immunochemical fecal occult blood test (FIT)-positive participants, enrolled in the Norwegian CRCbiome study during 2017–2021, aged 55–77 years. Absolute and energy-adjusted intakes of red and processed meat (combined and individually) were assessed using a validated, semi-quantitative FFQ. Associations between meat intake and screen-detected colorectal lesions were examined using multinomial logistic regression analyses with adjustment for key covariates. Of 1162 participants, 319 presented with advanced colorectal lesions at colonoscopy. High *v*. low energy-adjusted intakes of red and processed meat combined, as well as red meat alone, were borderline to significantly positively associated with advanced colorectal lesions (OR of 1·24 (95 % CI 0·98, 1·57) and 1·34 (95 % CI 1·07, 1·69), respectively). A significant dose–response relationship was also observed for absolute intake levels (OR of 1·32 (95 % CI 1·09, 1·60) per 100 g/d increase in red and processed meat). For processed meat, no association was observed between energy-adjusted intakes and advanced colorectal lesions. A significant positive association was, however, observed for participants with absolute intake levels ≥ 100 *v*. < 50 g/d (OR of 1·19 (95 % CI 1·09, 1·31)). In summary, high intakes of red and processed meat were associated with presence of advanced colorectal lesions at colonoscopy in FIT-positive participants. The study demonstrates a potential role of dietary data to improve the performance of FIT-based screening.

Colorectal cancer (CRC) represents a major global health burden, accounting for about one-tenth of all cancers diagnosed and cancer-related deaths each year^(1)^. The significant contribution to cancer mortality, together with the worrying rise in incidence seen globally^(2)^, highlights the need for identifying novel prevention strategies that are both feasible and effective at a large scale.

Diet is one of the major modifiable risk factors of CRC^(3–5)^. Typically, a Western dietary pattern, characterised by high amounts of red and processed meat, has been linked to increased disease risk^(6)^. Altering dietary habits have the potential to greatly reduce morbidity and premature mortality from CRC^(7)^. However, it is well known that achieving sustained dietary changes is difficult^(8)^. Thus, in order to obtain the desired cancer preventive effects, complementary prevention strategies are needed.

Screening with removal of precancerous lesions represents such a prevention strategy and has been shown to reduce both CRC incidence^(9–12)^ and mortality^(9–16)^. However, current screening methods have limitations. The most widely used screening method today is the fecal immunochemical test (FIT) for occult blood^(17)^. Despite being able to detect most CRC, a substantial proportion of the precancerous lesions (∼65–75 %) is not detected^(18,19)^, representing a missed opportunity given the preventive effect of removing these lesions. A further drawback of the FIT test is the suboptimal specificity, resulting in a high number of participants unnecessarily being referred for follow-up colonoscopy^(8)^.

To improve FIT-based screening, there has been a growing interest in developing risk scores aimed at predicting advanced colorectal lesions with higher accuracy than what is possible with FIT testing alone^(20,21)^. The integration of easy-to collect risk factor information into prediction algorithms represents a particularly attractive option given the expected ease and low costs associated with implementation. An important first step towards the development of such prediction algorithms is establishing risk factors for colorectal precancerous lesions. While substantial evidence exists regarding risk and protective factors of CRC^(3,4,22)^, less is known when it comes to the precancerous lesions, especially for the dietary factors where studies often have been compromised by the use of low-quality assessment tools^(23,24)^. In the present study, we aimed to examine the role of red and processed meat consumption – as major dietary risk factors for CRC^(3,25)^ – in early-stage colorectal carcinogenesis, using data from a large cohort of FIT-positive participants.

The primary aim of the study was to examine associations between intake of red and processed meat (combined and individually) and the presence of screen-detected non-advanced and advanced colorectal lesions at follow-up colonoscopy. Secondary aims were to examine whether potential associations detected differed by age, sex and adherence to cancer prevention recommendations and present positive predictive values (PPV) of the FIT test for the presence of advanced colorectal lesions at colonoscopy across the various meat consumption groups.

## Methods

### Bowel Cancer Screening in Norway and the CRCbiome study

The CRCbiome study is a prospective cohort study nested within the Bowel Cancer Screening in Norway (BCSN) trial, a pilot for an upcoming national screening programme^(26)^. The BCSN has a randomised trial design, comparing once-only sigmoidoscopy with repeated FIT tests every second year for up to four rounds. Since 2012, 139 291 women and men aged 50–74 years at enrolment, living in South East Norway, have been invited to participate. Of these, 70 096 have been recruited to the FIT arm, with a cumulative participation rate for the first three rounds of 68 %^(26)^.

During 2017–2021, the CRCbiome study recruited FIT-positive participants from the BCSN trial, with the aim of developing a microbiome-based classifier for improved detection of advanced colorectal lesions at screening^(27)^. Participants were invited after being informed about their FIT screening result, but before attending follow-up colonoscopy. With the invitation letter, participants received two questionnaires to be completed prior to the colonoscopy examination: a lifestyle and demographics questionnaire and a FFQ. Returning at least one of the questionnaires was regarded as consent to the study. Of 2698 participants invited to the study, 1653 agreed to participate, giving a participation rate of 61 %.

Both the BCSN and the CRCbiome study have been approved by the Regional Committee for Medical Research Ethics in South East Norway (Approval no.: 2011/1272 and 63148, respectively). The BCSN is also registered at clinicaltrials.gov (Clinical Trial (NCT) no.: 01538550).

### Study sample

**Fig. 1.**
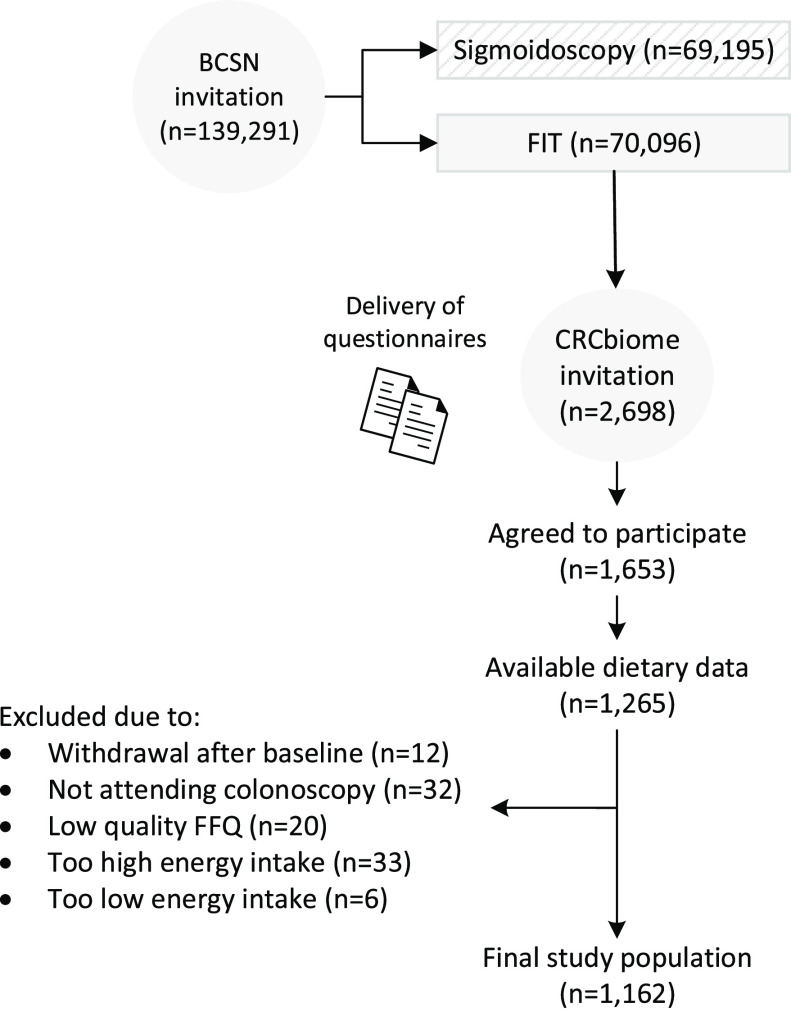
Flow chart of study participants. BCSN, Bowel Cancer Screening in Norway; FIT, fecal immunochemical test.

The current study included participants from the CRCbiome study with available dietary information by autumn 2021 (*n* 1265). After excluding participants who had withdrawn from the study after baseline (*n* 12), not attended colonoscopy (*n* 32), had a poor quality FFQ (*n* 20) or reported too low (< 2.5 MJ (600 kcal) and < 3.3 MJ (800 kcal) per day for women and men, respectively, *n* 6) or too high (> 14.6 MJ (3500 kcal) and > 17.6 MJ (4200 kcal) per day for women and men, respectively, *n* 33) energy intake (standard energy cut-off values were set according to Willett^(28)^), a final number of 1162 were eligible for the study (see flow chart, Fig. 1).

### Assessment of dietary intake, including red and processed meat

Dietary data were obtained using a self-administered semi-quantitative, 14-page FFQ, designed to capture the habitual diet including alcoholic beverages during the past year. The questionnaire is a modified version of an FFQ developed by the Department of Nutrition, University of Oslo^(29–35)^, which has been validated for a variety of nutrients^(29,31,34,35)^ and food groups^(31–35)^, including red and processed meat^(35)^. The questionnaire covers a total of 256 food and beverage items, of which five concern cold cuts and twenty-eight are meat-containing dishes. For each food item, participants are asked to record frequency of consumption during the preceding year, ranging from never/seldom to several times a day, and/or amount, typically as portion size given in various household units. Daily meat intake was calculated using the dietary calculation system KBS (short for ‘**K**ost**b**eregnings**s**ystem’), developed at the Department of Nutrition, University of Oslo. The most recent database, AE-18, was used. AE-18 is an extended version of the official Norwegian Food Composition Table, version 2018^(36)^. In the present paper, red meat intake was categorised into the following three groups: (1) ‘red meat’, including unprocessed meat from mammals, such as beef, veal, pork, lamb and goat, (2) ‘processed meat’, including red meat processed in any way intended to improve flavour or preservation (also the addition of salt as for minced meat and minced meat products) and (3) ‘red and processed meat’, being the sum of red and processed meat.

Prior to analyses, all questionnaires were reviewed and evaluated by trained personnel according to a standardised framework for quality control assessment developed by the study group^(27)^.

### Outcome assessment

Outcome data were obtained from the BCSN database, containing detailed clinicopathological information on all colorectal lesions detected at follow-up colonoscopy. The information was recorded by the responsible gastroenterologist using a structured recording system. Based on the findings of the colonoscopy report, participants were categorised into the following diagnostic groups: no adenoma, non-advanced adenoma and advanced colorectal lesions, the latter including both advanced adenomas (any adenoma with villous histology, high-grade dysplasia or adenoma diameter ≥ 10 mm), advanced serrated lesions (any serrated lesion with size ≥ 10 mm or dysplasia) and CRC (any adenocarcinoma of the colon or rectum)^(37)^. In cases of multiple findings, the most severe finding formed the basis for the outcome classification.

### Assessment of covariates

Lifestyle and demographic information were obtained using a self-administered, four page questionnaire, which was piloted in a targeted population prior to study start and adjusted according to participants’ feedback. The questionnaire includes ten questions in total, where the ones relevant to the current study included: demographic factors (national background, education, occupation and marital status), family history of CRC, diagnosis of chronic bowel disorders or food intolerance, smoking and snus habits and physical activity level. In the question concerning national background, participants were asked to select the geographic area best matching their parents’ country of birth. Participants selecting either ‘Norway’ or ‘North or Central Europe (outside of Norway), North America or Australia’ were referred to as ‘Western’, whereas participants selecting either ‘South Europe, South- or Central America’, ‘Asia’ or ‘Africa’ were referred to as ‘Non-Western’. With regard to tobacco usage, participants were asked about their current habits, including the daily number of cigarettes/snus portions, and to recall years since possible cessation and total years of use. In the present study, smokers and snusers were defined as self-reported regular or occasional users, or having quit consumption within the last 5 years. For physical activity, participants were asked to report the time spent in low, moderate and vigorous physical activity per week during the past year. The reply options were ‘never’ and six alternatives for activity in hours per week: ‘less than 0·5’, ‘0·5–1’, ‘1·5–2’, ‘2·5–3·5’, ‘4–6’ and ‘more than 7’. Total amount of moderate to vigorous physical activity (min/week) was calculated by summing the time spent in moderate and vigorous activity, the latter weighted by a factor of two to best match national^(38)^ and international physical activity guidelines^(39,40)^. For each reply option, the mid-interval value was used as basis for the calculation. BMI was calculated based on self-reported weight (kg) and height (cm) obtained from the FFQ.

To get an overall measure of the lifestyle habits of the participants, an index for adherence to the WCRF/AICR Cancer Prevention Recommendations of 2018 was made. The index is designed to measure adherence to the following seven cancer prevention recommendations: ‘have a healthy body weight’, ‘be physically active’, ‘eat a diet rich in wholegrains, vegetables, fruit, and beans’, ‘limit consumption of “fast foods” and other processed foods high in fat, starches and sugars’, ‘limit consumption of red and processed meat’, ‘limit consumption of sugar-sweetened drinks’ and ‘limit alcohol consumption’. For the six recommendations included (the recommendation on red and processed being excluded), a score of 0, 0·5 or 1 point was given for not complying, partly complying or fully complying to the recommendation, respectively, in line with the standardised scoring system proposed by Shams-White *et al*.^(41,42)^ Thus, the score ranged from zero to six points.

### Statistical analyses

Descriptive statistics are given as median (p25, p75) and numbers (percentages) for continuous and categorical variables, respectively.

To study correlations between the different meat variables (i.e. red and processed meat combined and individually, as well as different subtypes of processed meat) and total energy intake, Spearman’s correlation coefficients, *r* ^s^, were computed.

Multinomial logistic regression analyses were used to calculate the OR and 95 % CI for the presence of non-advanced and advanced colorectal lesions (relative to having no adenomas) by category of meat intake.

To calculate energy-adjusted meat intakes (i.e. the effect of substituting meat with other energetic sources to maintain the same energy intake), the ‘nutrient residual model’ was applied. With this approach, participants’ energy intake is adjusted indirectly by obtaining the residuals from linear regression models, with energy intake as independent variable and the different meat variables as dependent variables^(43)^. These residuals are then used in the further analyses.

The study population was divided into tertiles and quartiles based on participants’ meat consumption relative to total energy intake (i.e. the residuals obtained from the various linear regression models). To enable comparisons with other studies, the study population was also divided into exposure groups based on absolute intake levels according to commonly applied cut-off values (i.e. < 100 *v*. ≥ 100 g/d for red and processed meat and < 50 *v*. 50–99 and ≥ 100 g/d for processed meat)^(3)^. A comparison of the absolute intake-based and the residual intake-based categorisation is depicted in online Supplementary Fig. S1. Linear trends were examined by recoding the categorical variables into numerical ones (i.e. 1, 2 and 3 for the exposure variables divided into tertiles). The absolute intake variables were also examined on a continuous scale (i.e. per 100 and 50 g/d increase in intake of red and processed meat combined and processed meat alone, respectively).

A total of four adjustment sets were tested in the multinomial logistic regression analyses (see online Supplementary Table S1 for a complete overview). Only two of these were included in the main tables: an age- and sex-adjusted model and a fully adjusted model, the latter including the following covariates: age (continuous), sex, BMI (continuous), smoking status (smoker, non-smoker, missing), education level (primary school, high school, college/university, missing), family history of CRC (yes, no, unknown), nationality (Western, non-Western, missing), screening centre (centre 1 and 2) and a modified WCRF/AICR index (the subcomponents BMI and meat intake being subtracted)^(41)^. The covariates were selected based on *a priori* knowledge on the relationship between meat intake and colorectal carcinogenesis^(3–5)^. BMI was included as a confounder rather than a mediator, because of the cross-sectional design of the study.

To study potential effect modifications, stratified analyses by age (< 65 or ≥ 65 years), sex and lifestyle were performed. As basis for the lifestyle interaction analysis, a modified WCRF/AICR index was used. Compared with the modified version used in the adjustment set described above, only the point for adhering to the meat recommendation was subtracted from the score. A cut-off value of 3·5 points, corresponding to the median value, was used to categorise participants as having a healthy or unhealthy lifestyle. Statistical interactions were evaluated by performing likelihood ratio tests, comparing models with and without the respective interaction terms.

To examine the potential for bias, sensitivity analyses were conducted for the main analyses, restricting the sample set to those who had completed the FFQ prior to colonoscopy, those with a high quality FFQ (compared with the main analyses, which also included those with medium quality FFQ) and those without a self-reported or clinician-diagnosed bowel disorder.

As an exploratory analysis, the PPV of a positive FIT test for the presence of advanced colorectal lesions was computed for those with the lowest, moderate and highest energy-adjusted intake of red and processed meat in their diet. Statistical differences in PPV were examined using *χ* ^2^ tests.

In line with the most recent statement from the American Statistical Association on *P*-values^(44)^, emphasis was put on effect sizes, variation and uncertainty of the data rather than *P*-values in the interpretation of the results.

All statistical analyses were performed using RStudio, version 3.6.3 (The R Foundation for Statistical Computing).

## Results

### Key characteristics of the study population by meat intake

Table 1 shows the key characteristics of the study population by energy-adjusted intakes of red and processed meat. The different consumption groups were characterised by distinct demographic and lifestyle characteristics. Compared with the low and moderate consumers, those in the high consumption group were slightly younger, more likely to be male, less likely to present with a family history of CRC and less likely to have completed higher education. The high consumption group was also characterised by a greater proportion of tobacco users, a lower proportion adhering to physical activity guidelines, higher BMI values and a higher alcohol intake. The median number of days from filling out the questionnaires (FFQ and LDQ) to the colonoscopy was 5–7 d across the meat consumption groups.

**Table 1.** Key characteristics of the study population by tertiles of energy-adjusted intake of red and processed meat (*n* 1162)*

	Tertiles (T) of energy-adjusted red and processed meat intake					
Variables	T1 (*n* 388)		T2 (*n* 387)		T3 (*n* 387)	
Age, years	67·0 (60·8, 72·1)		67·8 (63·1, 72·3)		66·2 (61·0, 71·5)	
Male sex	187	48·2	207	53·5	265	68·5
Screening centre						
Centre 1 (Moss)	178	45·9	206	53·2	219	56·6
Centre 2 (Bærum)	210	54·1	181	46·8	168	43·4
Nationality						
Western	368	94·8	358	92·5	359	92·8
Non-Western	8	2·1	7	1·8	8	2·1
Missing	12	3·1	22	5·7	20	5·2
Family history of CRC						
Yes	75	19·3	64	16·5	57	14·7
No	283	72·9	293	75·7	279	72·1
Unknown	30	7·7	30	7·8	51	13·2
Education						
Primary school	53	13·7	73	18·9	78	20·2
High school	132	34·0	152	39·3	167	43·2
University/college	198	51·0	155	40·1	130	33·6
Missing	5	1·3	7	1·8	12	3·1
Marital status						
Married/cohabiting	282	72·7	312	80·6	313	80·9
Not married/non-cohabiting	102	26·3	68	17·6	63	16·3
Missing	4	1·0	7	1·8	11	2·8
Working status						
Employed	143	36·9	124	32·0	122	31·5
Retired/unemployed	241	62·1	255	65·9	253	65·4
Missing	4	1·0	8	2·1	12	3·1
Bowel disorder						
Yes	143	36·9	124	32·0	122	31·5
No	241	62·1	255	65·9	253	65·4
Unknown	4	1·0	8	2·1	12	3·1
Food intolerance						
Yes	55	14·2	58	15·0	50	12·9
No	261	67·3	260	67·2	273	70·5
Unknown	72	18·6	69	17·8	64	16·5
Smoking status						
Smoker	60	15·5	79	20·4	90	23·3
Non smoker	323	83·2	300	77·5	284	73·4
Missing	5	1·3	8	2·1	13	3·4
Snus status						
Snuser	14	3·6	19	4·9	29	7·5
Non-snuser	351	90·5	345	89·1	331	85·5
Missing	23	5·9	23	5·9	27	7·0
Alcohol intake, g/d	9·4 (2·1, 18·6)		7·3 (1·8, 17·6)		10·5 (2·8, 21·6)	
BMI, kg/m^2^	25·5 (23·1, 28·0)		26·4 (24·1, 29·0)		27·8 (25·3, 30·2)	
Physical activity level						
< 150 min/week	172	44·3	193	49·9	222	57·4
≥ 150 min/week	212	54·6	187	48·3	154	39·8
Missing	4	1·00	7	1·8	11	2·8
Time of FFQ relative to colonoscopy, days	–6 (–13, −1)		–5 (–12, −1)		–7 (–13, −1)	

CRC; colorectal cancer; T, tertile.

* Values are median (p25, p75) for continuous variables and *n* (%) for categorical variables.

### Daily intake of red and processed meat in the study population as a whole

Daily intake of the various meat types is provided in Table 2. The median intake of red and processed meat combined was 70 g/d (p25, p75:48–99 g/d), most of which were processed (median (p25, p75): 48 (30–70) g/d). For red meat, the median (p25, p75) intake was 20 (11–33) g/d, leading to the majority of participants (97 %) adhering to the WCRF/AICR recommendation on limiting consumption to < 500 g/week (i.e. 71 g/d). The rather high consumption of processed meat resulted in only 2 % adhering the WCRF/AICR recommendation on limiting consumption to < 21 g/week (i.e. 3 g/d)^(41)^. The main source of processed meat was minced meat products, with a median of 22 g/d (p25, p75:12–35 g/d). All meat variables were right-skewed, indicating the presence of a few high consumers for each meat category. Only 1 % of the study population reported no consumption of red or processed meat at all. Meat intake increased by energy intake (*r* ^s^ of 0·47 for red and processed meat combined).

**Table 2.** Daily intake of red and processed meat (g/d) in the study population as a whole (*n* 1162). Values represent absolute intake levels

Type of meat	Adherent to guidelines†		Zero consumers		Percentiles				Histogram	Correlation with energy
	*n*	%	*n*	%	25	50	75	100		*r*_s_ *
**Absolute intakes**										
Red and processed meat	593	51	8	1	48	70	99	345	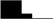	0·47*
Red meat	1130	97	29	2	11	20	33	148	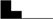	0·34*
Processed meat	21	2	12	1	30	48	70	251	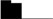	0·42*
Minced meat products	–		51	4	12	22	35	190	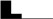	0·35*
Sausages	–		240	21	3	9	21	119	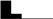	0·29*
Cold cuts	–		80	7	7	11	20	101	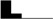	0·26*

r_s_, Spearman’s correlation coefficient.

* < 0·001.

† According to the proposed operationalisation of the Cancer Prevention Recommendations by WCRF/AICR of 2018, recommending limiting the consumption of red meat to < 500 g/week and processed meet to < 21 g/week^(40,41)^.

### Associations of red and processed meat intake with screen-detected colorectal lesions

Associations between energy-adjusted intakes of red and processed meat and colorectal lesions are shown in Table 3 (an extended version is given in online Supplementary Table S1).

**Table 3.** The presence of non-advanced and advanced colorectal lesions by energy-adjusted intakes of red and processed meat (*n* 1162)*

	Colonoscopy outcome									
	Meat intake (g/d)†		Non-advanced adenomas (*n* 411, 35 %)				Advanced lesions (*n* 319, 27 %)			
	Median	p25, p75	Age and sex adjusted		Multivariable adjusted‡		Age and sex adjusted		Multivariable adjusted‡	
			OR	95 %	OR	95 %	OR	95 %	OR	95 %
Energy-adjusted intakes										
Red and processed meat										
T1	41	0, 105	Ref		Ref		Ref		Ref	
T2	66	21, 140	1·03	0·74, 1·44	0·96	0·75, 1·24	1·17	0·82, 1·67	1·24	0·98, 1·58
T3	113	50, 345	1·26	0·90, 1·77	1·20	0·93, 1·54	1·27	0·88, 1·83	1·24	0·98, 1·57
*P*_trend_			0·19		0·31		0·21		0·27	
Red meat										
T1	8	0, 29	Ref		Ref		Ref		Ref	
T2	20	3, 43	0·91	0·65, 1·27	0·82	0·64, 1·05	1·20	0·83, 1·72	1·17	0·93, 1·47
T3	39	18, 148	0·95	0·68, 1·33	0·88	0·69, 1·13	1·38	0·96, 1·98	1·34	1·07, 1·69
*P*_trend_			0·76		0·46		0·09		0·12	
Processed meat										
T1	26	0, 70	Ref		Ref		Ref		Ref	
T2	47	12, 97	1·12	0·80, 1·56	1·09	0·86, 1·39	0·84	0·59, 1·20	0·86	0·67, 1·09
T3	81	39, 251	1·26	0·90, 1·78	1·21	0·95, 1·54	1·11	0·77, 1·59	1·06	0·84, 1·33
*P*_trend_			0·19		0·29		0·60		0·77	

p, percentile; Ref, reference; T, tertile.

* OR and 95 % CI are obtained from multinomial logistic regression analysis using two different adjustment sets: an age- and sex-adjusted model (n 1162) and a fully adjusted model (*n* 1158).

† Values represent absolute intake levels.

‡ Complete adjustment set: age (continuous), sex, energy intake (continuous), BMI (continuous), smoking status (smoker, non-smoker, missing), education level (primary school, high school, collage/university, missing), family history of CRC (yes, no, unknown), nationality (Western, non-Western, missing), screening centre (centre 1 and 2) and a modified WCRF/AICR score for adherence to cancer prevention recommendations (the subcomponents BMI and meat intake being subtracted).

For both the sum of red and processed meat and red meat alone, positive associations were observed between energy-adjusted intake levels and the presence of advanced colorectal lesions (Table 3). Compared to those with the lowest intake of red and processed meat (median of 41 g/d), those with an intermediate (median of 66 g/d) and high intake (median 113 g/d) both had a 24 % higher chance of presenting with advanced colorectal lesions (OR for T2 and T3 of 1·24 (95 % CI 0·98, 1·58) and 1·24 (95 % CI 0·98, 1·57), respectively). Having an intermediate (median of 20 g/d) or high (median of 39 g/d) intake of red meat was also associated with presenting with advanced colorectal lesions compared with having a low intake (median of 8 g/d), with OR of 1·17 (95 % CI 0·93, 1·47) and 1·34 (95 % CI 1·07, 1·69) for T2 and T3, respectively. The positive associations observed for high intake levels were confirmed in a supplementary analysis dividing the exposure variables into quartiles instead of tertiles (online Supplementary Fig. S2), as well as when studying absolute intake levels (Table 4, online Supplementary Table S2). Despite positive associations, no linear trends were observed in the analyses of energy-adjusted intakes and advanced colorectal lesions (Table 3). A significant dose–response relationship was, however, observed for absolute intake of red and processed meat (OR of 1·32 (95 % CI 1·09, 1·60) per 100 g/d increase). In contrast to what was observed for red meat, no association was observed between energy-adjusted intakes of processed meat and advanced colorectal lesions (OR of 1·06 (95 % CI 0·84, 1·33) comparing those with the highest (median of 81 g/d) to those with the lowest (median of 26 g/d) intake, Table 3). A positive association was, however, observed for the relatively small group of participants (*n* 111) with absolute intake levels ≥ 100 g/d relative to those with intakes below 50 g/d (OR of 1·19 (95 % CI 1·09, 1·31), Table 4). Of note, this group consisted almost solely of male participants (88 %). No associations were observed between moderate consumption levels of processed meat and advanced colorectal lesions (Table 3, Table 4, online Supplementary Table S2 and Supplementary Fig. S2).

**Table 4.** The presence of non-advanced and advanced colorectal lesions by absolute intakes of red and processed meat (*n* 1162)* (Odds ratios and 95 % confidence intervals)

	Colonoscopy outcome											
	Meat intake				Non-advanced adenoma (*n* 411, 35 %)				Advanced lesions (*n* 319, 27 %)			
Meat variables	Median†	p25, p75	*n*	%	Age and sex adjusted		Multivariable adjusted‡		Age and sex adjusted		Multivariable adjusted‡	
					OR	95 %	OR	95 %	OR	95 %	OR	95 %
Red and processed meat												
< 100 g/d	58	0, 100	876	75·4	Ref			Ref	Ref			Ref
≥ 100 g/d	130	100, 345	286	24·6	1·02	0·73, 1·42	1·02	0·84, 1·24	1·33	0·94, 1·88	1·15	0·96, 1·39
Per 100 g/d increase					1·03	0·74, 1·44	1·02	0·84, 1·24	1·53	1·09, 2·15	1·32	1·09, 1·60
Processed meat												
< 50 g/d	31	0, 50	603	51·9	Ref			Ref	Ref			Ref
50–99 g/d	67	50, 100	448	38·6	1·11	0·83, 1·50	1·11	0·86, 1·43	0·99	0·71, 1·36	0·88	0·69, 1·14
≥ 100 g/d	120	100, 251	111	9·6	0·97	0·57, 1·64	1·00	0·94, 1·06	1·45	0·87, 2·43	1·19	1·09, 1·31
Per 50 g/d increase					1·06	0·86, 1·31	1·07	0·86, 1·35	1·26	1·01, 1·57	1·14	0·91, 1·44

p, percentile; Ref, reference.

* OR and 95 % CI are obtained from multinomial logistic regression analysis using two different adjustment sets: an age- and sex-adjusted model (*n* 1162) and a fully adjusted model (*n* 1158).

† Values represent absolute intake levels.

‡ Complete adjustment set: age (continuous), sex, energy intake (continuous), BMI (continuous), smoking status (smoker, non-smoker, missing), education level (primary school, high school, collage/university, missing), family history of CRC (yes, no, unknown), nationality (Western, non-Western, missing), screening centre (centre 1 and 2) and a modified WCRF/AICR score for adherence to cancer prevention recommendations (the subcomponents BMI and meat intake being subtracted).

For the association analyses of red and processed meat intake with non-advanced lesions, no clear pattern could be detected (Tables 3 and 4, online Supplementary Table S2 and Supplementary Fig. S2).

### Stratified analyses by age group, sex and lifestyle

To examine the potential for divergent findings by age, sex and lifestyle habits, measured by a modified WCRF/AICR score (meat intake being subtracted), stratified analyses were performed. Analyses were restricted to the main meat variables (i.e. energy-adjusted red and processed meat, red meat and processed meat) and presented for the advanced colorectal lesions only (online Supplementary Fig. S3). By visually inspecting the forest plots, associations of red and processed meat with advanced colorectal lesions appeared stronger for the male participants, those older than 65 years of age and those with an unhealthy lifestyle. However, formal statistical testing did not confirm these factors as effect modifiers of the meat-advanced colorectal lesion relationship (all likelihood ratio test *P*-values > 0·05, online Supplementary Fig. S3).

### Sensitivity analyses

When restricting the analyses to those with high quality FFQ (*n* 1149); those who had delivered the questionnaire prior to becoming aware of their colonoscopy result (*n* 1051); those without any self-reported or confirmed chronic bowel disorders (*n* 979) or those without a self-reported or confirmed inflammatory bowel disease (*n* 1115), only modest fluctuations in effect estimates were observed (online Supplementary Fig. S4). The sensitivity analyses largely confirmed the results obtained using the complete data set. High energy-adjusted intakes of red and processed meat and red meat alone remained positively associated with the presence of advanced colorectal lesions with OR ranging from 1·24 to 1·33 and 1·31 to 1·40, respectively, when comparing the upper with the lower tertile. For the association of energy-adjusted intake of processed meat with advanced colorectal lesions, the OR comparing the highest to the lowest tertile ranged from 1·07 to 1·15.

### Red and processed meat intake and performance of the fecal immunochemical test

**Fig. 2.**
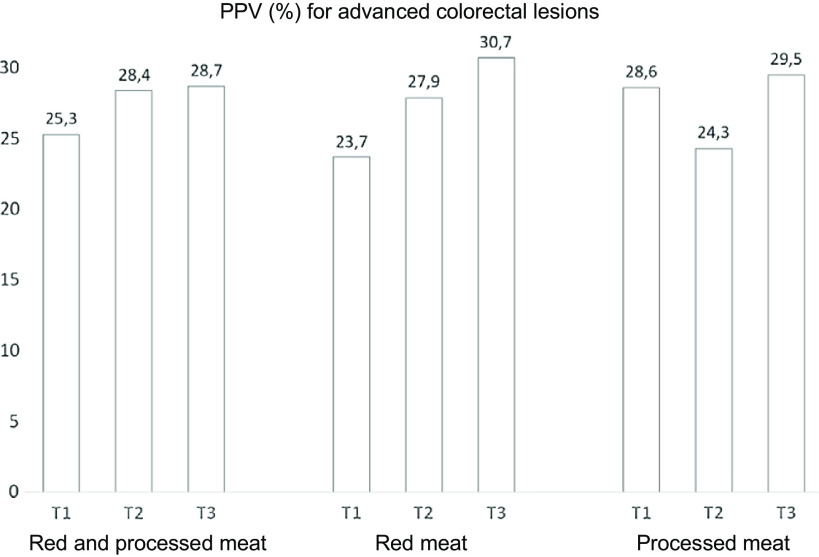
Positive predictive values (PPV) of the positive FIT test for the presence of advanced colorectal lesions among participants with the lowest (T1), medium (T2) and highest (T3) energy-adjusted intakes of red and processed meat.

To examine the potential of using meat consumption data to improve the performance of FIT-based screening, PPV for the presence of advanced colorectal lesions were calculated for the different meat consumption groups (Fig. 2). Although no statistically significant differences could be detected, a tendency towards higher PPV was observed for the groups of participants with medium to high energy-adjusted intakes of red and processed meat combined and red meat alone compared to those with the lowest intakes. For processed meat, no clear pattern could be detected.

## Discussion

In this cross-sectional investigation among FIT-positive participants, high intakes of red and processed meat were associated with increased probability of presenting with advanced colorectal lesions at follow-up colonoscopy. The strongest associations were observed for those having high energy-adjusted intakes of red and processed meat (largely being driven by red meat), as well as among those with a particularly high absolute intake of processed meat (≥ 100 g/d). Having a moderate consumption of processed meat was not associated with the presence of colorectal lesions relative to eating low amounts.

The positive associations observed between medium to high intakes of red meat and the presence of advanced colorectal lesions are in line with a systematic review and meta-analysis by Aune *et al*. from 2013, showing a 29 % increased risk of advanced adenoma per 100 g increment in red meat intake per day^(45)^. Together, these results coincide with the literature on CRC. In the latest review and meta-analysis of the WCRF/AICR from 2017, a 12 % risk increase was observed per 100 g of red meat consumed per day^(3)^. A significant positive association was also observed in a recent umbrella review of meta-analyses of prospective cohort studies by Veettil *et al*., examining a variety of dietary factors in relation to CRC incidence^(46)^. In this review, red meat intake (high *v*. low absolute intakes) came out as one of two dietary exposures with convincing evidence for an increased risk of CRC. The evidence remained robust after various sensitivity analyses, including the exclusion of effect estimates not adequately controlled for confounding.

In contrast to what was observed for red meat, no associations were observed between energy-adjusted intakes of processed meat and the presence of advanced colorectal lesions. We did, however, observe a positive association (OR of 1·19) for the small male-dominated proportion of participants with particularly high absolute intake levels (≥ 100 g/d). In the systematic review and meta-analysis by Aune *et al*, the summary relative risk for advanced adenoma was 1·29 per 50 g of processed meat consumed per day^(45)^. With regard to CRC, the review and meta-analysis of WCRF/AICR from 2017 found a 16 % increased risk of CRC per 50 g of processed meat consumed per day^(3)^. A significant positive association between high intakes of processed meat and CRC risk was also observed in the umbrella review of meta-analyses by Veettil *et al.* ^(46)^, as well as a recent systematic review of prospective cohort studies by Händel *et al.* ^(50)^ However, in both these reviews – where different criteria for quality evaluation were applied – risk of bias in the included studies was considered high. A major concern was inadequate control for confounding, being particularly worrisome given the close connection between processed meat intake and other CRC risk promoting behaviours^(51)^ – also being evident in the present study. Whether the discrepancy in results between ours and the aforementioned studies is due to differences in adjustment sets (ours in general including more covariates) is unclear. Other potential explanations include differences in controlling for energy intake, distribution and categorisation of the exposure variable, as well as how the definition of processed meat was operationalised. In the present study, we chose to limit processed meat to that of mammals (i.e. excluding poultry) – a practice which is common in many, but not all studies. We also defined minced meat as being processed, as the majority of minced meat sold in Norway is added salt. Although these decisions may have influenced our results, the magnitude is likely small given the low consumption of white processed meat in the population (only raising the median for processed meat with 2 g/d), as well as the low intake of pure minced meat (median of 6 g/d). Aside from the methodological issues discussed, there may be true differences in risk in the populations studied.

In many epidemiological studies, including the previously mentioned review and meta-analyses of WCRF/AICR^(3)^, increased CRC risk has been observed at intake levels of about 100 and 50 g/d for red and processed meat, respectively. Based on this, WCRF/AICR has recommended limiting the consumption of red meat to < 350–500 g/week and consuming as little processed meat as possible (typically operationalised as < 21 g/week). Although our findings support the existing cancer prevention recommendation to limit consumption of red meat, our study does not support the strict recommendation for processed meat. For none of the approaches used to study processed meat intake, associations between moderate consumption levels and the presence of advanced colorectal lesions were detected. Taken together with the high risk of bias noted in the recent reviews of processed meat and CRC, this challenges the concept that no lower level of processed meat in the diet is safe (1). Given people’s values and preference towards processed meat consumption^(52)^ – also evident by the low adherence to the recommendation observed in the present study (2 %), this warrants further investigation to clarify the cancer risk associated with including moderate amounts of processed meat in the diet.

The evidence linking high meat consumption to CRC risk is largely based on prospective cohort studies with repeated assessment of the dietary intake. Interestingly, we observed that even recent meat intake, assessed at the time of CRC screening, predicted the presence of advanced colorectal lesions at follow-up colonoscopy. Although not significant, the PPV of the positive FIT test for advanced colorectal lesions were higher in participants with the highest energy-adjusted intakes of red and processed meat combined and red meat alone. The predictive ability of meat and other dietary variables in a screening setting has also been noted by others^(53)^. The result points towards a potential role of including dietary information in future prediction algorithms aimed at improving early detection of CRC, including our own CRCbiome study^(27)^.

Several mechanisms have been suggested to explain the cancer-promoting effects of red and processed meat^(54)^. First, red and processed red meat contain several potential carcinogens^(3,54,55)^. Some are naturally present in meat (e.g. heme iron, being particularly enriched in red meat), whereas others are added during the industrial processing (e.g. nitrates and nitrites) or formed as a result of high temperature cooking (e.g. heterocyclic amines and polycyclic aromatic hydrocarbons). Second, red and processed meat may promote cancer development through complex interactions with the gut microbiome. In addition to being involved in the metabolism of potentially carcinogenic compounds, bioactive molecules derived from the fermentation of red meat proteins – of which 10 % is assumed to reach the colon^(56)^ – such as hydrogen sulfide, ammonia, secondary bile acids and phenolic compounds, have been linked to increased CRC risk^(57)^. It has also been suggested that the microbial state of the individual, being shaped by a multitude of environmental and lifestyle exposures, may modify the response to red and processed meat intake. In the present study, we observed a tendency towards effect estimates being stronger for those with an unhealthy lifestyle. This could indicate that host factors, including but not limited to the gut microbiome, dictate how the individual responds to high amounts of meat in the diet. Potential interactions between meat consumption and the microbial state of the individual on early-stage colorectal carcinogenesis will be addressed in a follow-up study of the present investigation.

Major strengths of this study include its large sample of FIT-positive participants (319 (27 %) being diagnosed with advanced colorectal lesions), use of a validated semi-quantitative FFQ to assess dietary intake and access to detailed information on likely confounders of the relationship between meat and advanced colorectal lesions. Furthermore, this study had access to clinically verified outcome data, minimising the chances of misclassification bias. A feature separating this study from most prior investigations is the approach used to adjust for total energy intake (i.e. the ‘nutrient residual model’). This allowed us to compare groups of individuals separated by their relative contribution of meat in the diet rather than absolute intake levels alone, having been suggested to increase power when the exposure variable is categorised^(43,58)^.

The study also has some limitations. Firstly, the participation rate of 68 % in the FIT screening, of which 61 % filled out the questionnaires mandatory to join the present study, may have resulted in selection bias. In a recent registry-based study characterising nonparticipants of CRC screening, we showed that participation in FIT screening was lower among those with lower socio-economic status, immigrant background and certain chronic diseases. Compliance to the follow-up colonoscopy in FIT positive was also lower among those with immigrant background, long driving time to the screening centre, as well as those with certain chronic diseases^(59)^. Second, exclusive selection of FIT-positive participants in the CRCbiome study may have limited the generalisability of the findings. However, as physiological bleeding is a common cause of a positive test result^(60)^, we consider it likely that the identified associations are relevant also to the screening population as a whole. A third limitation relates to the dietary assessment method used in the study. As for any instrument used to measure dietary habits, the FFQ is prone to measurement errors, which may have influenced our results. However, various measures were taken to mitigate these errors, including the exclusion of low-quality questionnaires prior to analysis, as well as the conduction of post-hoc sensitivity analyses. A fourth limitation relates to the cross-sectional design, preventing causal interpretations. The findings of this study should therefore be followed up by prospective cohort studies or randomised controlled trials.

In summary, in this high-risk group of CRC screening participants – all being FIT-positive, high intakes of red and processed meat were associated with the presence of advanced colorectal lesions at follow-up colonoscopy. The strongest associations were observed for those with the highest energy-adjusted intakes of red and processed meat (largely being driven by red meat), as well as for those with a particularly high absolute intake of processed meat. Having a moderate consumption of processed meat did not seem to impose any risk. The largest cancer preventive effects could likely be achieved by targeting individuals with the highest consumption of red and processed meat in their diet as part of a holistic approach to cancer prevention. The potential added benefit of incorporating dietary variables into risk scores to improve the diagnostic accuracy of FIT deserves further investigation.

## Supplementary material

For supplementary material accompanying this paper visit https://doi.org/10.1017/S0007114522002860. click here to view supplementary material
