# Supplementary material for: Associations of red and processed meat intake with screen-detected colorectal lesions
Source: Br J Nutr. 2022 Sep 7;129(12):2122–32. doi: 10.1017/S0007114522002860 (PMC10197083; doi:10.1017/S0007114522002860)
Supplement: Supplementary file 1 [file S0007114522002860sup.zip › S0007114522002860sup001.docx]

**Supplementary information**

| **Supplementary Table 1.** Odds ratios (ORs) and 95% confidence intervals (CIs) for presence of non-advanced and advanced colorectal lesions by energy-adjusted intakes of red and processed meat (n=1,162). All adjustment sets are included (models 1-4)^1^. | | | |
| --- | --- | --- | --- |
|  |  | **Colonoscopy outcome** | |
|  | **Meat intake (g/day)**  Median (p25, p75)^2^ | **Non-advanced adenoma**  (n=411, 35%) | **Advanced lesions**  (n=319, 27%) |
| **Energy-adjusted intakes** | |  |  |
| Red and processed meat |  |  |  |
| T1 | 41 (0, 105) | Ref | Ref |
| T2 | 66 (21, 140) |  |  |
| *Model 1* |  | 1.03 (0.74, 1.44) | 1.17 (0.82, 1.67) |
| *Model 2* |  | 1.01 (0.78, 1.29) | 1.28 (1.01, 1.64) |
| *Model 3* |  | 0.98 (0.77, 1.26) | 1.24 (0.98, 1.57) |
| *Model 4* |  | 0.96 (0.75, 1.24) | 1.24 (0.98, 1.58) |
| T3 | 113 (50, 345) |  |  |
| *Model 1* |  | 1.26 (0.90, 1.77) | 1.27 (0.88, 1.83) |
| *Model 2* |  | 1.24 (0.98, 1.58) | 1.33 (1.05, 1.68) |
| *Model 3* |  | 1.19 (0.93, 1.52) | 1.24 (0.98, 1.58) |
| *Model 4* |  | 1.20 (0.93, 1.54) | 1.24 (0.98, 1.57) |
| Red meat | |  |  |
| T1 | 8 (0, 29) | Ref | Ref |
| T2 | 20 (3, 43) |  |  |
| *Model 1* |  | 0.91 (0.65, 1.27) | 1.20 (0.83, 1.72) |
| *Model 2* |  | 0.89 (0.70, 1.14) | 1.28 (1.02, 1.59) |
| *Model 3* |  | 0.86 (0.67, 1.09) | 1.24 (0.99, 1.56) |
| *Model 4* |  | 0.82 (0.64, 1.05) | 1.17 (0.93, 1.47) |
| T3 | 39 (18, 148) |  |  |
| *Model 1* |  | 0.95 (0.68, 1.33) | 1.38 (0.96, 1.98) |
| *Model 2* |  | 0.94 (0.74, 1.20) | 1.42 (1.14, 1.77) |
| *Model 3* |  | 0.89 (0.70, 1.14) | 1.35 (1.08, 1.70) |
| *Model 4* |  | 0.88 (0.69, 1.13) | 1.34 (1.07, 1.69) |
| Processed meat | |  |  |
| T1 | 26 (0, 70) | Ref | Ref |
| T2 | 47 (12, 97) |  |  |
| *Model 1* |  | 1.12 (0.80, 1.56) | 0.84 (0.59, 1.20) |
| *Model 2* |  | 1.11 (0.88, 1.39) | 0.89 (0.71, 1.12) |
| *Model 3* |  | 1.12 (0.88, 1.42) | 0.87 (0.69, 1.09) |
| *Model 4* |  | 1.09 (0.86, 1.39) | 0.86 (0.67, 1.09) |
| T3 | 81 (39, 251) |  |  |
| *Model 1* |  | 1.26 (0.90, 1.78) | 1.11 (0.77, 1.59) |
| *Model 2* |  | 1.25 (0.99, 1.58) | 1.16 (0.92, 1.45) |
| *Model 3* |  | 1.23 (0.97, 1.57) | 1.09 (0.86, 1.38) |
| *Model 4* |  | 1.21 (0.95, 1.54) | 1.06 (0.84, 1.33) |

*^1^Odds ratios (ORs) and 95% confidence intervals (CIs) are obtained using multinomial logistic regression models adjusted for the following covariates: Model 1: age (continuous) and sex (n=1162), model 2: model 1 covariates and energy intake (continuous) (n=1162), model 3: model 2 covariates, BMI (continuous), smoking status (smoker, non-smoker, missing) and education level (primary school, high school, collage/university, missing) (n=1158), and model 4: model 3 covariates, family history of CRC (yes, no, unknown), nationality (western, non-western, missing), screening center (center 1 and 2) and a modified WCRF/AICR score for adherence to cancer prevention recommendations (the subcomponents BMI and meat intake being subtracted) (n=1158).*

*^2^Values represent absolute intake levels.*

*Abbreviations: CI; confidence interval, OR; odds ratio, p; percentile, Ref; reference, T; tertile.*

| **Supplementary Table 2.** Odds ratios (ORs) and 95% confidence intervals (CIs) for presence of non-advanced and advanced colorectal lesions by absolute intakes of red and processed meat (n=1,162). All adjustment sets are included (models 1-4)^1^. | | | |
| --- | --- | --- | --- |
|  |  | **Colonoscopy outcome** | |
|  | **Meat intake (g/day)**  Median (p25, p75)^2^ | **Non-advanced adenoma**  (n=411, 35%) | **Advanced lesions**  (n=319, 27%) |
| **Absolute intakes** |  |  |  |
| Red and processed meat |  |  |  |
| T1 | 37 (0, 55) | Ref | Ref |
| T2 | 70 (55, 89) |  |  |
| *Model 1* |  | 1.14 (0.82, 1.60) | 1.35 (0.93, 1.95) |
| *Model 2* |  | 1.17 (0.91, 1.50) | 1.29 (1.02, 1.64) |
| *Model 3* |  | 1.15 (0.89, 1.49) | 1.26 (1.00, 1.59) |
| *Model 4* |  | 1.13 (0.87, 1.46) | 1.22 (0.95, 1.56) |
| T3 | 116 (89, 345) |  |  |
| *Model 1* |  | 1.10 (0.77, 1.56) | 1.44 (0.98, 2.10) |
| *Model 2* |  | 1.16 (0.89, 1.50) | 1.30 (1.02, 1.64) |
| *Model 3* |  | 1.09 (0.84, 1.42) | 1.20 (0.95, 1.52) |
| *Model 4* |  | 1.08 (0.83, 1.41) | 1.19 (0.94, 1.50) |
| Red meat | |  |  |
| T1 | 8 (0, 14) | Ref | Ref |
| T2 | 20 (14, 28) |  |  |
| *Model 1* |  | 1.06 (0.76, 1.48) | 1.21 (0.84, 1.74) |
| *Model 2* |  | 1.07 (0.84, 1.36) | 1.16 (0.92, 1.45) |
| *Model 3* |  | 1.04 (0.81, 1.32) | 1.11 (0.88, 1.40) |
| *Model 4* |  | 1.01 (0.79, 1.29) | 1.07 (0.85, 1.35) |
| T3 | 40 (28, 148) |  |  |
| *Model 1* |  | 0.79 (0.56, 1.12) | 1.31 (0.91, 1.88) |
| *Model 2* |  | 0.80 (0.63, 1.03) | 1.21 (0.96, 1.51) |
| *Model 3* |  | 0.75 (0.58, 0.97) | 1.15 (0.91, 1.44) |
| *Model 4* |  | 0.75 (0.58, 0.97) | 1.16 (0.92, 1.46) |
| Processed meat | |  |  |
| T1 | 23.6 (0, 36) | Ref | Ref |
| T2 | 48 (36, 62) |  |  |
| *Model 1* |  | 1.12 (0.80, 1.57) | 1.31 (0.91, 1.88) |
| *Model 2* |  | 1.13 (0.89, 1.45) | 1.26 (1.00, 1.59) |
| *Model 3* |  | 1.11 (0.90, 1.37) | 1.21 (0.98, 1.49) |
| *Model 4* |  | 1.09 (0.85, 1.40) | 1.17 (0.92, 1.50) |
| T3 | 82 (62, 251) |  |  |
| *Model 1* |  | 1.09 (0.77, 1.54) | 1.14 (0.78, 1.67) |
| *Model 2* |  | 1.13 (0.88, 1.46) | 1.02 (0.80, 1.29) |
| *Model 3* |  | 1.10 (0.90, 1.35) | 0.95 (0.78, 1.16) |
| *Model 4* |  | 1.07 (0.83, 1.39) | 0.91 (0.72, 1.16) |

*^1^Odds ratios (ORs) and 95% confidence intervals (CIs) are obtained using multinomial logistic regression models adjusted for the following covariates: Model 1: age (continuous) and sex (n=1162), model 2: model 1 covariates and energy intake (continuous) (n=1162), model 3: model 2 covariates, BMI (continuous), smoking status (smoker, non-smoker, missing) and education level (primary school, high school, collage/university, missing) (n=1158), and model 4: model 3 covariates, family history of CRC (yes, no, unknown), nationality (western, non-western, missing), screening center (center 1 and 2) and a modified WCRF/AICR score for adherence to cancer prevention recommendations (the subcomponents BMI and meat intake being subtracted) (n=1158).*

*^2^Values represent absolute intake levels.*

*Abbreviations: CI; confidence interval, OR; odds ratio, p; percentile, Ref; reference, T; tertile.*


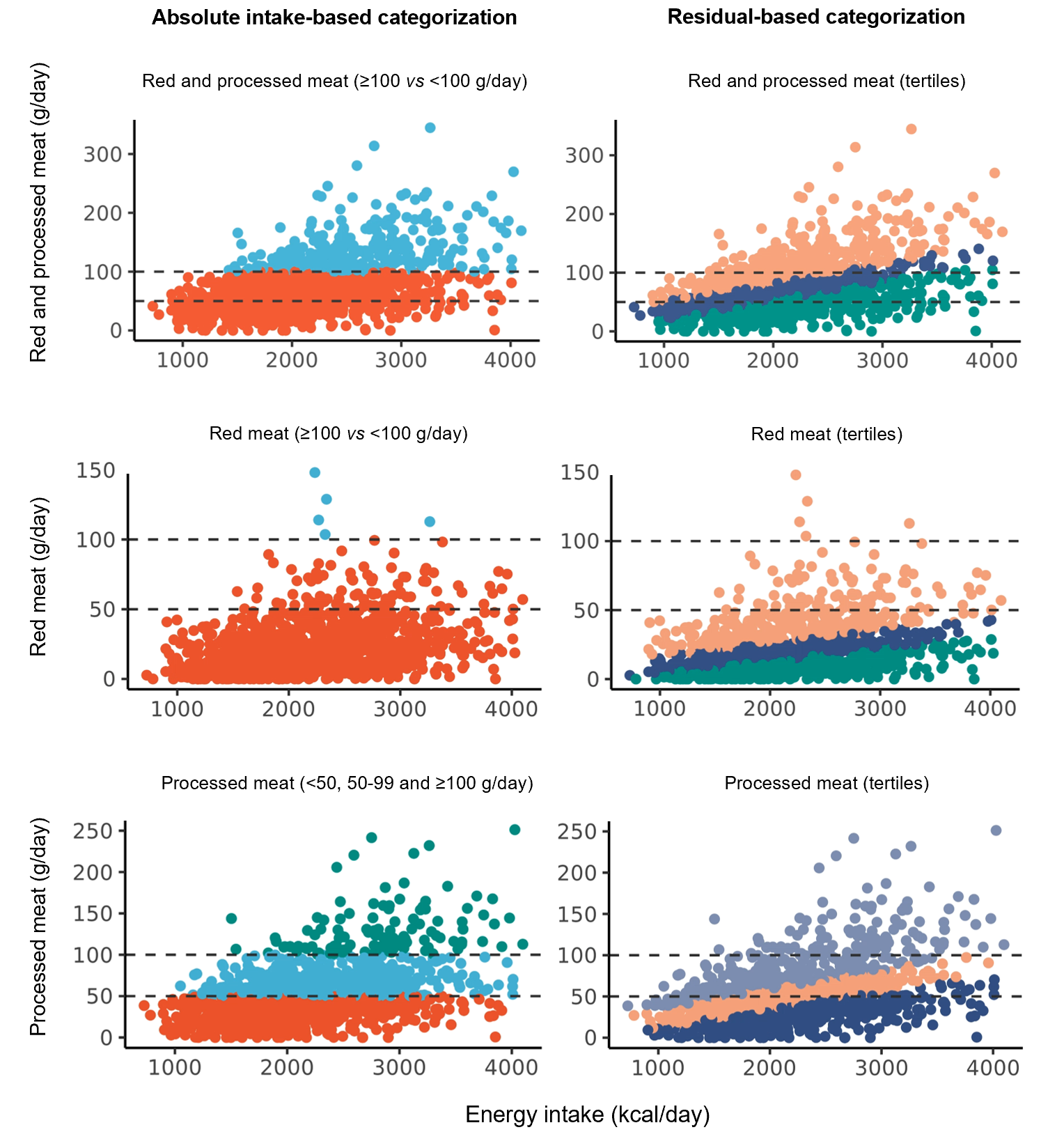


**Supplementary Figure 1**. Relationship between total energy intake (kcal/day) and meat consumption (g/day) with color indicating the category of the exposure variable the participant has been assigned to. Due to the few (n=5) individuals with red meat intake ≥ 100 g/day, no association analyses were performed for this dichotomized variable.


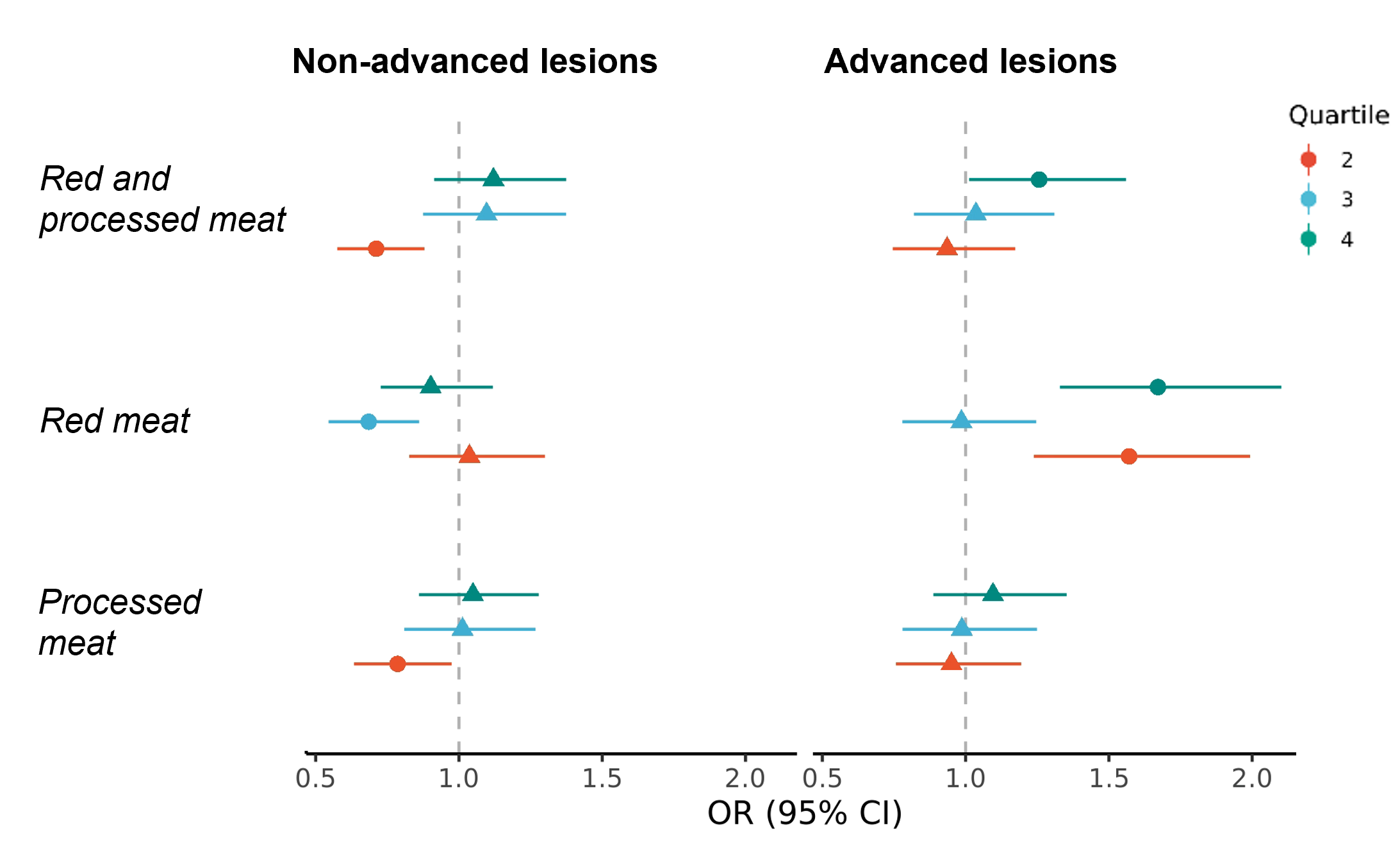


**Supplementary Figure 2**. Associations of energy-adjusted intakes of red- and processed meat with non-advanced and advanced colorectal lesions, using quartile split. Analyses are adjusted for the following covariates: age (continuous), sex, energy intake (continuous), BMI (continuous), smoking status (smoker, non-smoker, missing), education level (primary school, high school, collage/university, missing), family history of CRC (yes, no, unknown), nationality (western, non-western, missing), screening center (center 1 and 2) and a modified WCRF/AICR score for adherence to cancer prevention recommendations (the subcomponents BMI and meat intake being subtracted). The circle-shaped dots indicate statistically significant effect estimates (p<0.05). Abbreviations: CI; confidence interval, OR; odds ratio.


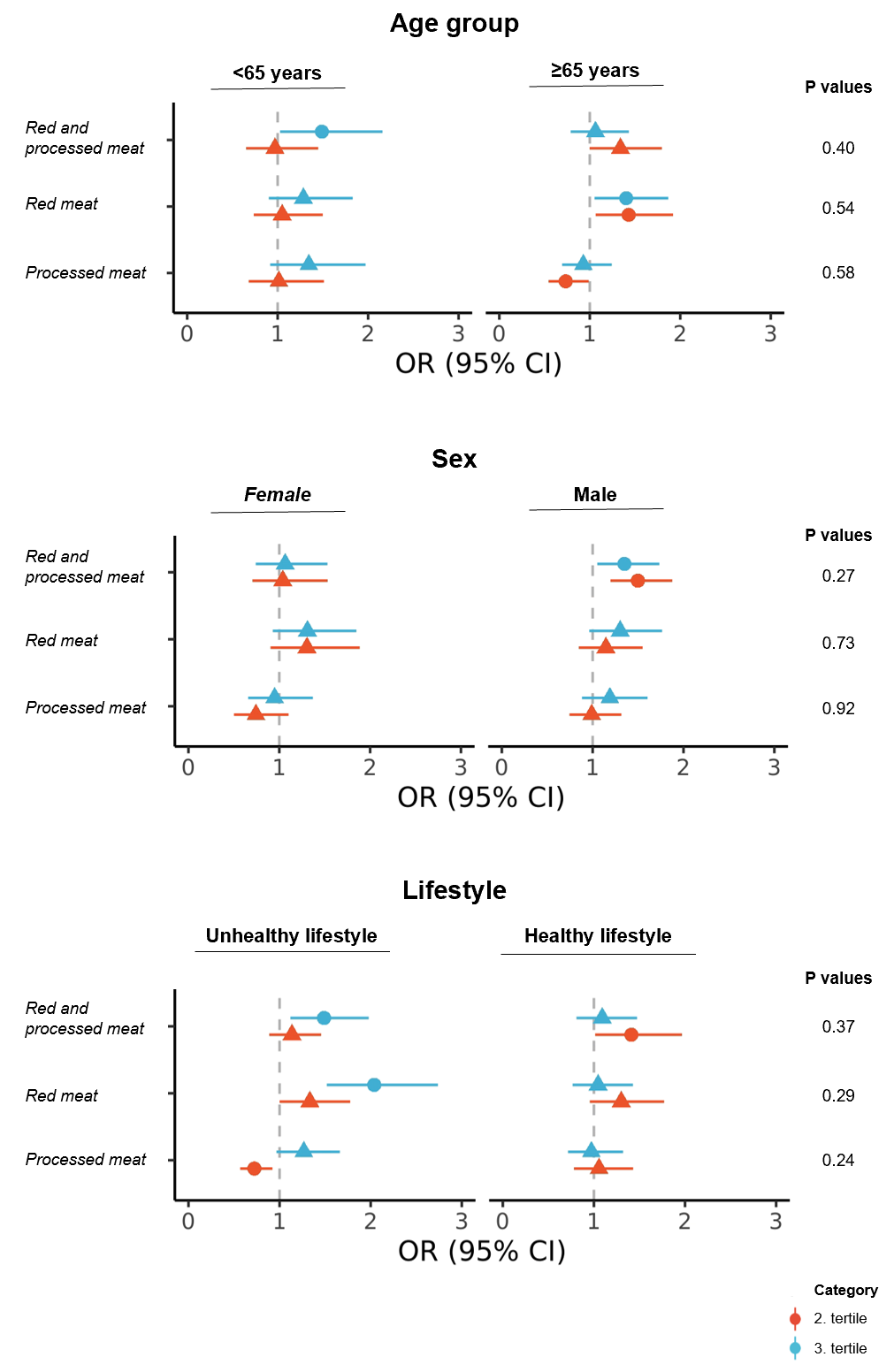


**Supplementary Figure 3**. Associations of energy-adjusted intakes of red and processed meat with advanced colorectal lesions, stratified by age group, sex and lifestyle, measured by adherence to the cancer prevention recommendations of 2018 from the WCRF/AICR. The WCRF/AICR index is a modified version of the original index, where the point for the meat recommendation has been subtracted. The adjustment sets of the respective models were as follows: 1) Age group: sex, energy intake (continuous), BMI (continuous), smoking status (smoker, non-smoker, missing) and education level (primary school, high school, collage/university, missing), 2) Sex: age (continuous), energy intake (continuous), BMI (continuous), smoking status (smoker, non-smoker, missing) and education level (primary school, high school, collage/university, missing), 3) Lifestyle: age (continuous), sex, energy intake (continuous), BMI (continuous), smoking status (smoker, non-smoker, missing) and education level (primary school, high school, collage/university, missing). The circle-shaped dots indicate statistically significant effect estimates (p<0.05). P values are derived from likelihood ratio tests, comparing the multinomial logistic regression models with and without the respective interaction terms. Abbreviations: CI; confidence interval, OR; odds ratio.


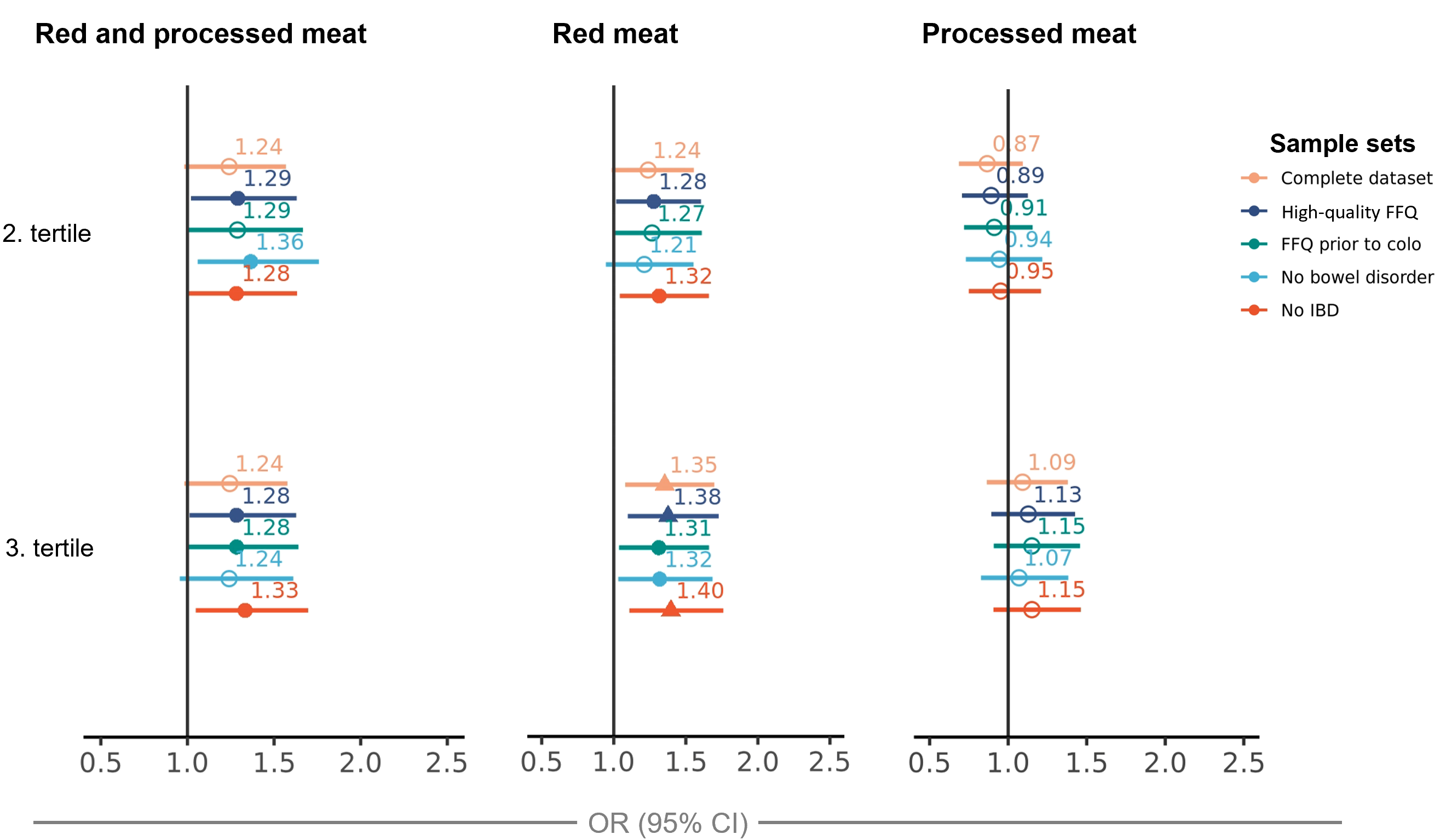


**Supplementary Figure 4**. Sensitivity analyses for the associations of energy-adjusted intakes of red and processed meat with advanced colorectal lesions in subsamples of the study population. All analyses are adjusted for the following covariates: age (continuous), sex, energy intake (continuous), BMI (continuous), smoking status (smoker, non-smoker, missing) and education level (primary school, high school, collage/university, missing). Whereas color filled effect estimates indicate significance, the shape of the effect estimate indicates level of significance (triangle: <0.01, circle: <0.05). Abbreviations: CI; confidence interval, colo; colonoscopy, FFQ; food frequency questionnaire, IBD; inflammatory bowel disease, OR; odds ratio.
